# A Smart Camera Trap for Detection of Endotherms and Ectotherms

**DOI:** 10.3390/s22114094

**Published:** 2022-05-28

**Authors:** Dean M. Corva, Nathan I. Semianiw, Anne C. Eichholtzer, Scott D. Adams, M. A. Parvez Mahmud, Kendrika Gaur, Angela J. L. Pestell, Don A. Driscoll, Abbas Z. Kouzani

**Affiliations:** 1 School of Engineering, Deakin University, Geelong, VIC 3216, Australia; d.corva@deakin.edu.au (D.M.C.); n.semianiw@deakin.edu.au (N.I.S.); scott.adams@deakin.edu.au (S.D.A.); m.a.mahmud@deakin.edu.au (M.A.P.M.); 2 School of Life and Environmental Sciences, Deakin University, Burwood, VIC 3125, Australia; aeichholtzer@deakin.edu.au (A.C.E.); kgaur@deakin.edu.au (K.G.); apestell@deakin.edu.au (A.J.L.P.); d.driscoll@deakin.edu.au (D.A.D.)

**Keywords:** animal behaviour, computerized monitoring, ectotherm, endotherm, environmental monitoring, image capture, image motion analysis, smart cameras

## Abstract

Current camera traps use passive infrared triggers; therefore, they only capture images when animals have a substantially different surface body temperature than the background. Endothermic animals, such as mammals and birds, provide adequate temperature contrast to trigger cameras, while ectothermic animals, such as amphibians, reptiles, and invertebrates, do not. Therefore, a camera trap that is capable of monitoring ectotherms can expand the capacity of ecological research on ectothermic animals. This study presents the design, development, and evaluation of a solar-powered and artificial-intelligence-assisted camera trap system with the ability to monitor both endothermic and ectothermic animals. The system is developed using a central processing unit, integrated graphics processing unit, camera, infrared light, flash drive, printed circuit board, solar panel, battery, microphone, GPS receiver, temperature/humidity sensor, light sensor, and other customized circuitry. It continuously monitors image frames using a motion detection algorithm and commences recording when a moving animal is detected during the day or night. Field trials demonstrate that this system successfully recorded a high number of animals. Lab testing using artificially generated motion demonstrated that the system successfully recorded within video frames at a high accuracy of 0.99, providing an optimized peak power consumption of 5.208 W. No water or dust entered the cases during field trials. A total of 27 cameras saved 85,870 video segments during field trials, of which 423 video segments successfully recorded ectothermic animals (reptiles, amphibians, and arthropods). This newly developed camera trap will benefit wildlife biologists, as it successfully monitors both endothermic and ectothermic animals.

## 1. Introduction

Camera trapping is the practice of using remotely triggered cameras for the automated gathering of images and/or videos of animals or other subjects passing in the camera’s field of view [1]. This non-invasive monitoring method has been a powerful tool for wildlife research over the past decade [1]. Camera trapping has been widely adopted for monitoring wildlife habitats and asset protection [2]. To successfully record an animal under motion, the camera trap needs a form of triggering to initiate a recording sequence. Triggering methods can include the use of passive infrared (PIR) sensors, seismic sensors, ultrasonic sensors, time-lapse, and artificial intelligence (AI). The most common triggering method utilizes a PIR sensor [3,4,5]. A custom Fresnel lens focuses infrared (IR) radiation to a pyroelectric sensor, allowing for the detection of rapid changes in background IR from a combination of heat and motion using thermally sensitive crystals inside the PIR sensor. This sensor then triggers the camera to commence recording [6,7,8].

Most current camera traps suffer from data loss through false negatives when an animal is present but not recorded, impacting data quality [6]. Most PIR-type sensors are known to produce false negatives [6]. These sensors can detect moving animals that have a 2.7 °C difference in temperature from the surrounding environment [2]. Ectothermic animals rarely produce temperatures differing 3 °C from the surrounding environment [9,10,11]. Thus, ectotherms are not reliably detectable using most PIR sensors [9].

Another form of triggering uses seismic sensors to convert motions into an electrical signal, initiating a recording sequence [12]. Seismic sensors are limited by high false alarm rates due to onboard data processing algorithms being unable to accurately discriminate different types of objects with light-weighted objects producing weak signals [12]. Digital seismic analysis commonly requires a lot of memory space for continuous recording and is a time-consuming process [13].

Ultrasonic sensors have been used as an alternative triggering method and offer fast response times. Using a transducer to output an ultrasonic acoustic wave, the reflected wave is measured by a secondary transducer to determine the proximity of an object using the Doppler effect [14]. The disadvantage of ultrasonic sensors is that the acoustic waveform falls within many animals’ audible hearing range, reducing the number of animals within the area [15].

Time lapse cameras are another well-known approach in camera trapping, where manual processing of footage is performed after recording is complete to reduce false-positives of no animals in recorded footage [16]. Some manual processing is also required to collect other relevant data (species, sex, etc.) [16].

Ecological forecasts often conclude that climate change is a high threat to ectotherms in the wild, as little is known about their potential for thermal adaptation [17]. Wildlife monitoring programs are used to indicate current numbers and the health of animals in the wild [18]. Camera trapping is a key tool in this research, as live footage identifies the species present and provides important behavioural insights, such as recent observations of invasive European wasps interfering with Dingo foraging [19].

Current wildlife camera traps using PIR sensors have some ability to detect ectothermic species under warm conditions [20,21]. The main aim of this research is to develop an advanced camera trap that is suitable for use in extreme outdoor environments with the ability to monitor ectotherms and endotherms. It is expected to improve wildlife monitoring capacity and ultimately contribute to more effective wildlife management and conservation.

In this work, we have implemented an AI algorithm as the triggering method of our camera trap. Previous attempts using AI have been made [22]; however, they do not focus on ectothermic activity. Sophisticated algorithms determine changes in video frames, allowing for the analytical evaluation of object tracking, motion detection, and pattern recognition for endotherms and ectotherms [23,24]. Current video surveillance cameras often adopt an AI algorithm as the triggering method for recording video, as in our proposed camera trap. However, the cameras generally have triggering thresholds for detecting humans and large objects at distant focal lengths; thus, they can potentially miss critical recordings from small animals and objects at short focal lengths. In addition, on-board environmental logging is not common on video surveillance cameras.

This paper is organized as follows. Section 2 provides an overview of the newly developed system. Section 3 describes the methods of experimentation for the camera trap. Section 4 describes the results of the newly developed system with discussions on its effectiveness. Section 5 highlights the limitations and how the system can be improved with future works. Finally, Section 6 concludes the work in the paper.

## 2. Camera Trap System Overview

**Figure 1:**
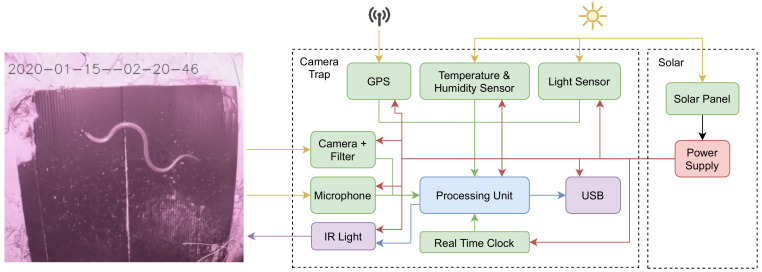
Hardware architecture of the camera trap.

**Figure 2:**
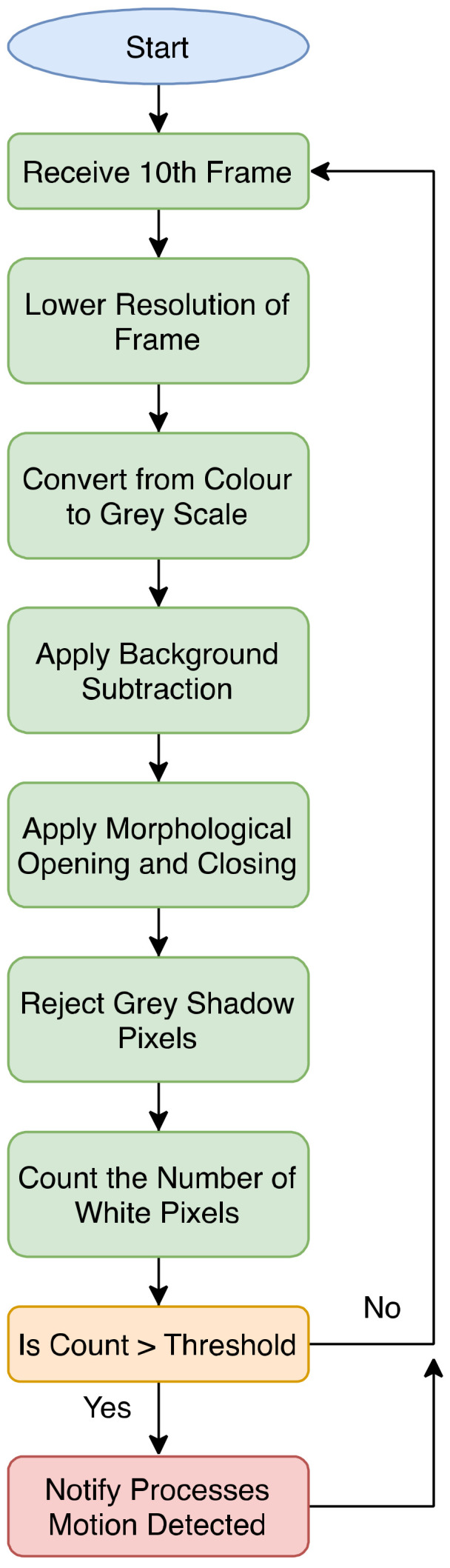
Motion detection algorithm.

**Figure A1:**
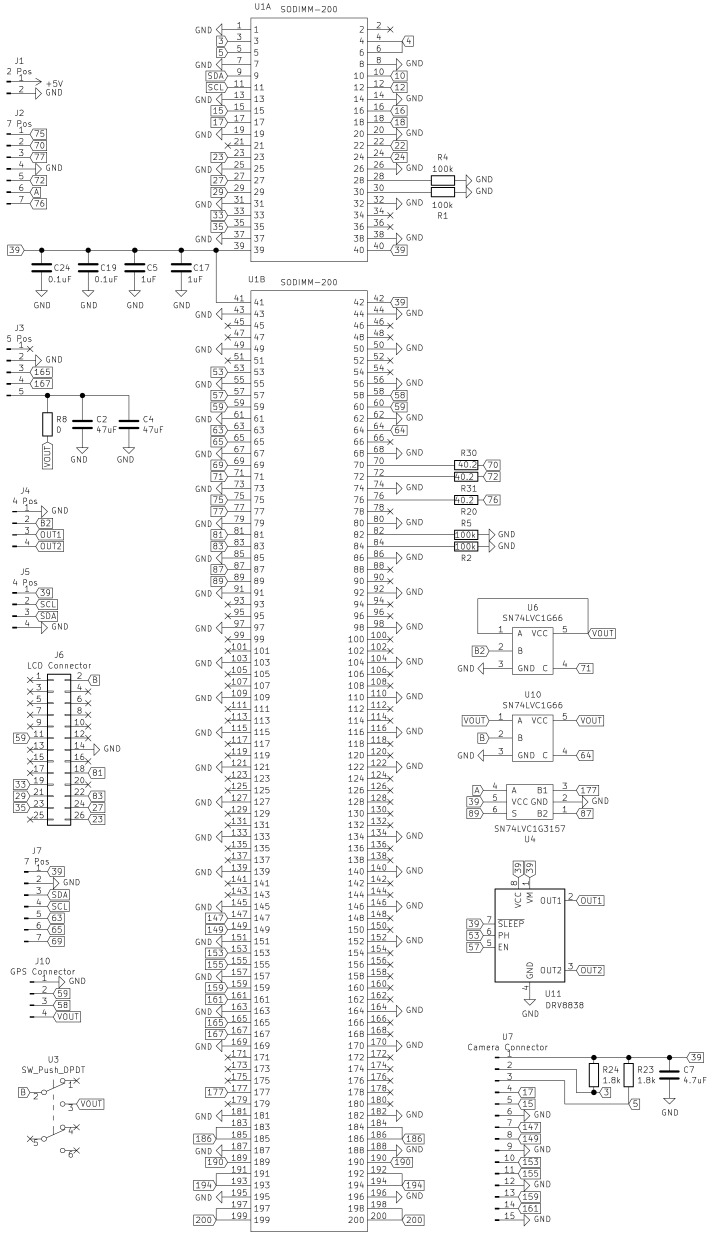
Electronics schematic of the camera trap.

**Figure A2:**
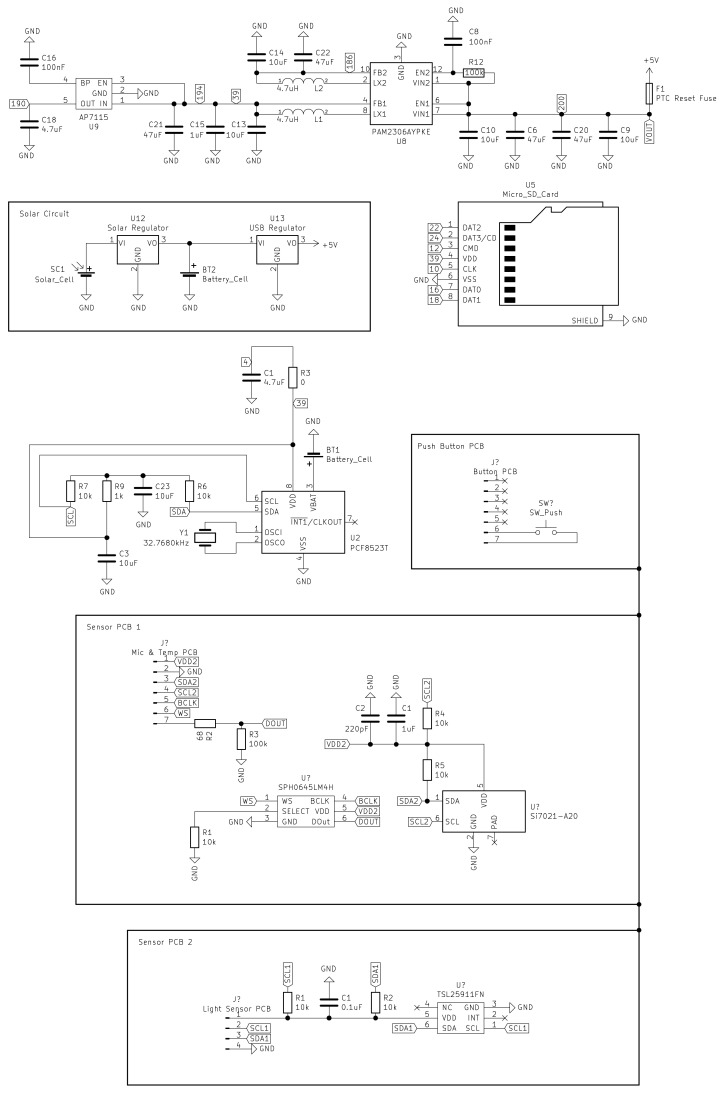
Electronics schematic of the camera trap.

The hardware architecture of the camera trap is shown in Figure 1. It has the ability to be self-sufficient, running on solar power when in operation. The camera trap uses an AI algorithm (Figure 2) as the triggering method. Features on the camera trap include global positioning system (GPS), temperature, humidity, and light sensing for environment and location data logging. Figure A1 and Figure A2 in Appendix A show the electronics schematic of the camera trap system.

### 2.1. Algorithm

Video data processing that uses AI algorithms can enable computers to better interpret the visual world [25]. Computers can attempt to identify a three-dimensional (3D) scene from two-dimensional (2D) images using a camera to gather digital images (in terms of the structure present in the scene) [25].

### 2.2. Foreground Detection

Foreground detection, also known as background subtraction, detects changes in image sequences and is used for motion detection and pattern recognition [26]. We chose a mixture of Gaussian (MOG) as the method of foreground detection for use in our device for its easy integration and development. MOG is a method that is composed of several distribution type Gaussians, each identified by k ∈ {1,…, *K*}, where *K* is known as the number of clusters in a dataset [27,28,29]. Gaussian distribution explains the data contained within each of the *K* clusters, where the mixing coefficients are probabilities and must meet Equation (1) [27]:

(1) $$\begin{array}{c} \overset{K}{\underset{k=1}{\sum }}\pi_{k}=1\\ K=Cluster \end{array}$$

The optimal values for Equation (1) are determined using the Gaussian density function, shown in Equation (2) [27]:

(2) $$\begin{array}{c} \rho \left(x|\mu ,\;\Sigma \right)=\frac{1}{{\left(2\pi \right)}^{\frac{n}{2}}\;{\left|\Sigma \right|}^{\frac{1}{2}}}\exp(-\frac{1}{2}{\left(x-\mu \right)}^{T}\Sigma^{-1}\left(x-\mu \right))\\ x=Data\;Points\\ n=Dimensions\;of\;each\;Data\;Point\\ \mu =Mean\\ \Sigma =Covariance \end{array}$$

### 2.3. Hardware

A single-board computer (SBC) was selected as the central processing unit (CPU) for the camera trap. The Raspberry Pi (RPi) computer module 3B+ was chosen for its wide availability and low cost. The RPi features a Broadcom BCM2837 system on a chip (SoC) with an integrated ARM-compatible CPU running at 1.4 GHz and an integrated graphics processing unit (GPU). It contains 1 GB of random-access memory (RAM), runs OpenCV, and allows for the use of a Micro Secure-Digital (micro-SD) card.

Four custom fabricated printed circuit boards (PCB) were manufactured for the hardware components of the camera trap. Components mounted to the main PCB included a DDR2 socket for mounting the RPi and a micro-SD card slot for the micro-SD card containing a Raspbian operating system (OS) running OpenCV. The camera module used was the Arducam 5-megapixel camera, which communicates directly to the RPi via a ribbon cable on the main PCB. An optional 3.5-inch display was also used during the configuration of the device, which communicates using SPI via a connector on the main PCB. The device uses a 256 GB external universal serial bus (USB) memory for writing video, audio, and sensor data files, which was wired to the main PCB. A GPS module was used to store the device’s current location and was also wired directly to the main PCB. An infrared (IR) filter was used for filtering unwanted IR light during the day. This is triggered by pulsing using the RPi’s general-purpose input and output (GPIO) pins, connected to the main PCB. An IR light (940 nm) was used to correctly operate the motion detection algorithm in low-light conditions, illuminating the capture area, and was wired directly to the main PCB. A real-time clock (RTC) was used to monitor local time on the RPi.

An SPH0645LM4H microphone using the I2S communication protocol was connected to the main PCB alongside SI7021 temperature and humidity sensors, which both communicate using I2C. Both sensors were mounted to an external PCB and connected to the main PCB using an interconnecting cable. A TSL2591 light intensity sensor communicates with I2C and was mounted on a separate PCB using an interconnecting cable to the main PCB. A final external PCB contained the power push button (see Figure A1 and Figure A2 for all listed components in the electronics schematic).

Before integrating all associated hardware, testing was conducted on individual components to identify any cross-communication or other errors. It was determined through testing that analysing every 10th frame would allow the camera to operate at 25 frames-per-second (fps). Analysing more frames would slow the fps, reducing recorded video quality.

All prior listed hardware was mounted in an ingress protection (IP) 67-rated aluminium enclosure. A solid 5 mm aluminium block was mounted using thermal tape to the CPU of the RPi to the enclosure for heat dissipation purposes. A transparent acrylic window was mounted to the aluminium lid of the enclosure to allow light to pass through to the light sensor. The enclosure featured an IP67-rated ON/OFF button for turning the RPi on and off. The USB memory was externally mounted and featured an IP67-rated cover. A custom faceplate was fabricated using black and clear acrylic to mount the camera, microphone, and IR light. A custom clear acrylic bracket was fabricated for inside the enclosure, where the PCB and camera module were mounted. Other external connections to the camera trap included an SMA connector for the GPS antenna and a power connector for the solar.

### 2.4. Solar

A solar system was used to power the camera trap. This solar system was composed of a 100-watt folding monocrystalline solar panel, 9-amp-hour (aH) sealed lead-acid (SLA) battery, solar charge controller, and an acrylonitrile butadiene styrene (ABS) enclosure. All necessary wiring was correctly rated for electrical specifications of the camera trap and outdoor ratings.

### 2.5. Software

**Figure 3:**
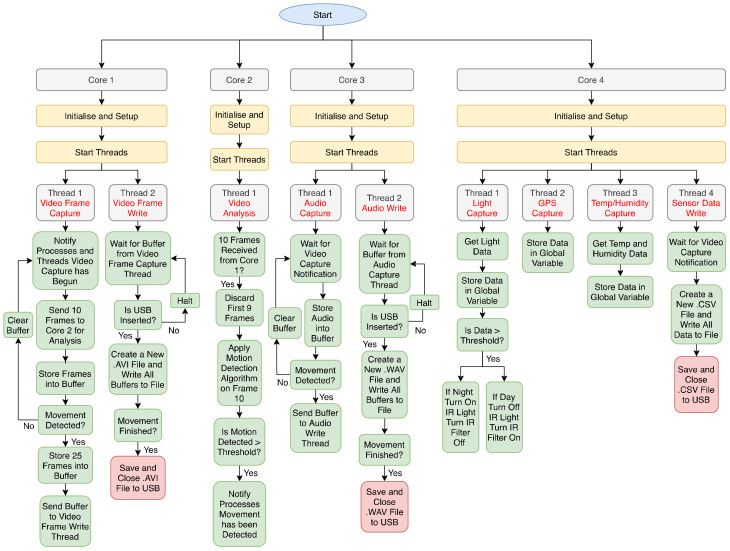
Software operation flowchart.

**Figure 4:**
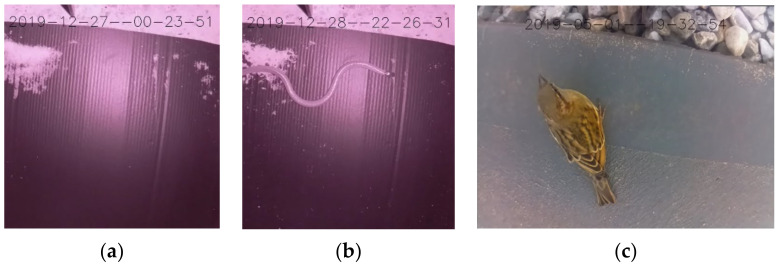
Footage example where (**a**) motion is not detected, (**b**) motion is detected, and (**c**) a bird is captured in good light conditions.

Software used in the camera trap first begins with initialization of peripherals and ports on all four cores. As the RPi contains four cores, the software designates appropriate tasks to each core. This is illustrated in Figure 3, the software flowchart. Core 1 is used for video frame capture and recording of video. Once the video frame capture has commenced, it notifies other threads and processes that video capture has begun. The first 10 frames are sent to Core 2, the video analysis core. Core 2 then begins its process of discarding the first 9 frames it receives and retaining the 10th frame, where it then applies the motion detection algorithm to this frame (see Figure 2 for motion detection algorithm). The motion detection algorithm first lowers the resolution of the 10th frame, converts it to greyscale, applies background subtraction to the frame, then applies morphological opening and closing to the frame where opening removes small objects from the background and closing removes small holes in the foreground, followed by removing grey shadow pixels, and finally counts the number of white pixels. The algorithm can then determine if motion has been detected by comparing the change in the number of white pixels with a threshold value. If motion has been detected, Core 2 alerts other cores to begin processes reliant on motion detection, including storing video frames into a buffer and sending the buffer to thread 2 of Core 1 (see Figure 4a,b for two examples of footage demonstrating frames that would and would not pass as detected motion). Figure 4c shows a bird captured in good light conditions.

The thread first checks if a USB memory is connected; otherwise, it will shut down the RPi. If connected, the thread creates an AVI file where the sent video buffers are stored. Once Core 2 determines that motion has stopped, thread 2 of Core 1 closes the AVI file and saves it to the USB memory. Core 3 is used for audio capture and storage of audio. Once Core 1 has alerted that video capture has begun, the audio capture thread stores audio into a buffer; if no motion is detected, the buffer is cleared. Otherwise, the buffer is sent to thread 2 of Core 3. Thread 2 checks if a USB memory is connected; otherwise, it will shut down the RPi. If connected, the thread creates a WAV file where the sent audio buffers are stored. Once Core 3 determines that motion has stopped, thread 2 of Core 3 closes the WAV file and saves it to the USB memory. Core 4 is used for data collection of sensors. Thread 1 is used for capturing the light sensor data. Once light sensor data have been captured, they are stored in a global variable of the RPi. If the light data are above a threshold, the RPi turns on the IR light and opens the IR filter (if low light conditions) or turns off the IR light and closes the IR filter (if good light conditions). Thread 2 of Core 4 captures GPS data and stores them in a global variable. Thread 3 of Core 4 captures the temperature and humidity data and stores them in a global variable. Thread 4 waits for Core 1 to alert that video capture has begun; then, it creates a CSV file where it stores all sensor data and saves them to the USB memory.

### 2.6. Interfacing

When a new USB memory is inserted into the camera trap, three files are created on it. These include a YAML file containing adjustable settings (good light and low light thresholds, RTC time), a text document describing how the camera operates, and a folder containing captured video, audio, and sensor data.

**Figure A3:**
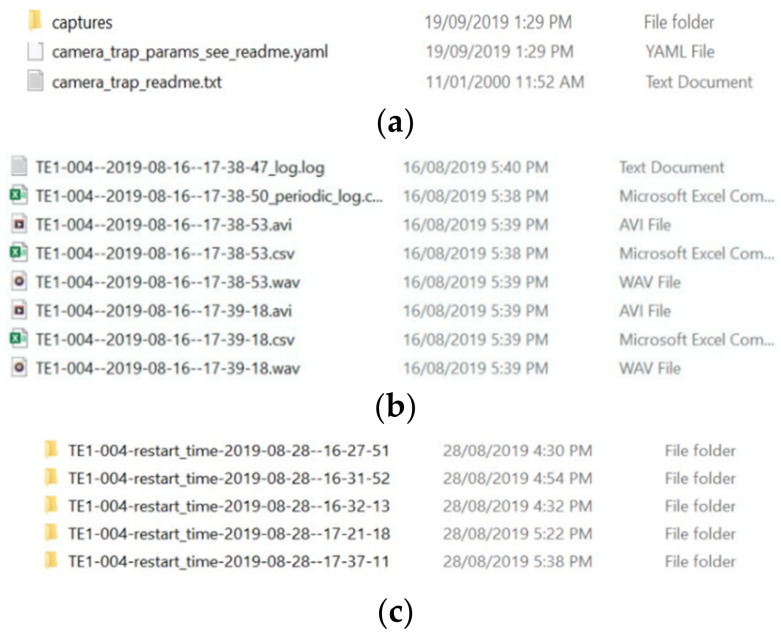
(**a**) File formats on USB memory, (**b**) contents of capture folder on USB, and (**c**) contents of restart folders on USB memory.

Figure A3a shows the file formats on the USB memory. The captures folder contains a new folder each time the camera trap is restarted, with the date and time of restart on the folder. This is shown in Figure A3b. A log document (in .txt format) to report errors occurring during operation, an excel file of logged sensor data, an AVI file of recorded video, and a WAV file for recorded audio are contained inside each restart folder. This is shown in Figure A3c.

### 2.7. Completed Camera Trap

**Figure 5:**
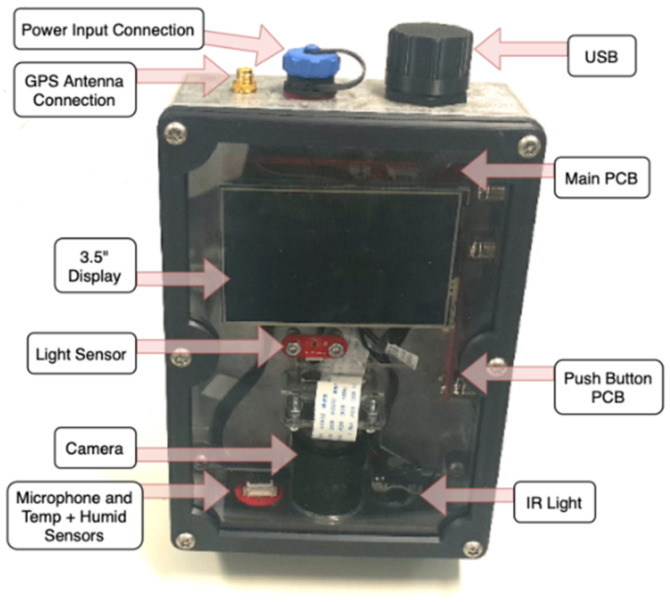
Completed camera trap.

The completed camera trap is presented in Figure 5, where portability and low cost were highest priority. The entire camera trap is compact, transported in a carry case including the solar setup. The 3.5-inch display can be removed, and the entire faceplate is constructed with clear acrylic for visibility of the display and light sensor.

### 2.8. Camera Trap Setup

**Figure 6:**
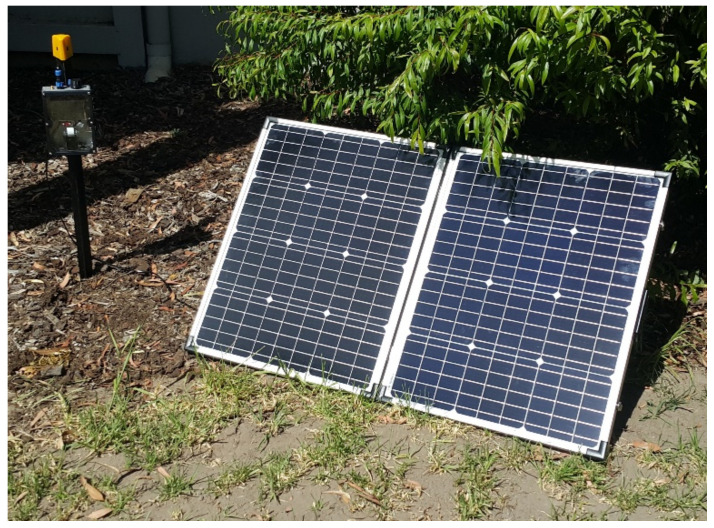
Camera trap setup consisting of camera trap mounted on stake, recording the ground, and solar panel powering the setup.

The camera trap was designed to be mounted to a stake vertically, where footage would be captured directly below the camera. The camera trap simply bolts to the stake, which is pre-driven into the ground. This ensures the camera trap is rigid for recording purposes. The solar panel is located nearby, at an angle of 45 degrees, to capture solar energy. The setup of the camera trap is illustrated in Figure 6.

The camera trap’s field of view is based on an image sensor measuring 2.74 mm × 3.76 mm with a focal length of 4 mm. Using a field of view of 300 × 300 mm, the mounting height of the camera is 319.14 mm. The lens is focused and then locked in place prior to sealing the case to ensure clear footage is recorded at the right scale for small ectotherms.

The detection threshold is calculated with a focal length of 300 × 225 mm, and the resolution of the camera is reduced from 1920 × 1080 to 640 × 480 to maintain the processing speed. Using the focal length and resolution, each pixel size is calculated to be 0.47 × 0.47 mm. Thus, the smallest animal that can be detected by the camera is 6 × 6 mm.

## 3. Methodology

### 3.1. Performance Evaluation

The overall performance of the developed camera trap system was evaluated by conducting three tests. Experiment 1 evaluated power consumption of the camera trap and localized footage testing. Experiment 2 was conducted to find true positives, true negatives, false positives, and false negatives from 43 videos using data obtained over 22 h in a lab environment, where artificial motion was created using a servo motor that moved a 3D-printed object of 5 × 2 × 3 cm 180 degrees once every 30 min in front of the camera trap to create 5-min segments of footage at a distance of 30 cm. Further testing of the camera trap was conducted over 19 days outdoors. Experiment 3 involved thermal testing using an environmental chamber while constantly recording. This involved leaving the camera trap inside the chamber with temperatures varying between 20 and 50 °C at a humidity between 50 and 100% to simulate Australian outdoor conditions. This was conducted to see if the CPU and enclosure were able to dissipate enough heat during operation under different temperature conditions.

### 3.2. In-Field Testing

**Figure 7:**
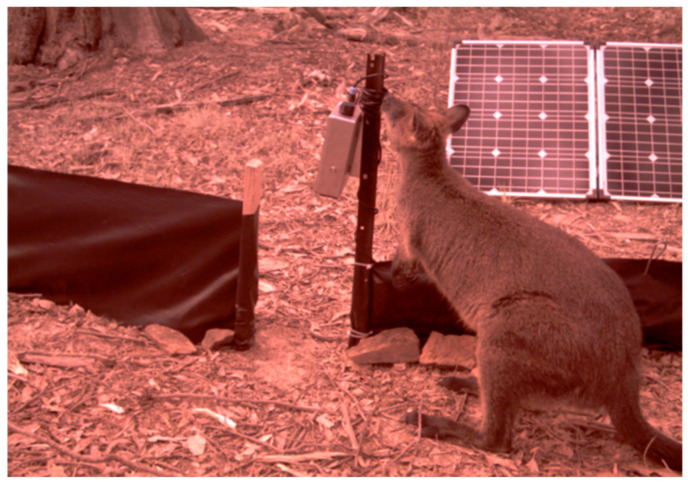
Camera trap setup mounted to stake with artificial passage, directing animals into the camera’s field of view of the ground, and solar panel powering the setup.

We installed 36 camera traps in remote locations of Little Desert National Park and Mt Arapiles-Tooan State Park, Victoria, Australia. These locations are dense with animals. However, they are difficult to access. Therefore, all the camera traps were left out for approximately 50 days, beginning on 5 December 2019. An artificial passage was created to better direct animals into the cameras field of view (see Figure 7). We set the cameras to be highly sensitive to movement so that the passage of small animals was not missed. The size of the detectable animal is programmable depending on the threshold set on the motion detection algorithm. We could increase and decrease the threshold; during our motion detection test, we could measure the size of 6 × 6 mm. After 50 days, the camera traps were collected and sent back to our laboratory for post-processing of data.

## 4. Results and Discussions

**Figure 8:**
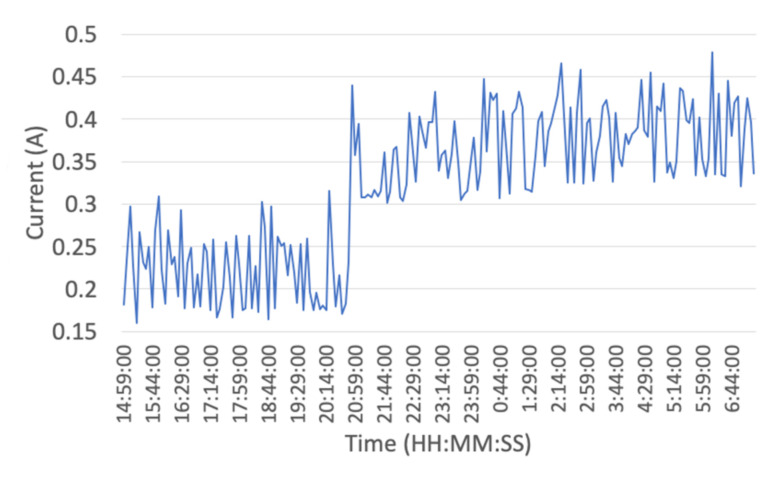
Power consumption of camera trap.

Experiment 1 examined the power consumption of the camera trap over 24 h, with 16 h of results shown in Figure 8. Due to the memory overflow of our datalogger, we could not access the rest of the results. A power increase occurs around 9 pm due to the camera trap entering night mode, where turning on the IR LED is done to illuminate the camera’s viewing area. Table 1 and Table 2 show the results of these tests with peak and average power and current consumption determined through good light conditions, where sunlight was present, and low light conditions, where sunlight was not present. Comparing these results to prior related works, previous attempts using similar components show an average current consumption of 230 mA [30]. In contrast, our system achieved an average of 221 mA current consumption under the same conditions, with even lower power consumption to be implemented in the future along with better optimization of hardware and software. Using a lower power LED could further reduce the power consumption; however, this may impact night-time image collection. The majority of the power consumption from our system was from writing files to the USB memory.

Using these data justifies the large 100 W solar panel and 9 Ah SLA battery, as low-light conditions may not charge the battery high enough in-field over extended operation time. Solar conditions also change year-round, cementing the decision for a large panel and battery limited by weight the operator can handle.

Experiment 2 resulted in calculating the accuracy and determining camera trap performance in lab conditions (Table 3). The definitions of the parameters presented in Table 3 can be found in reference [31].

Calculating the results from Experiment 2 using the confusion matrix, 264 5-min time segments were assumed in a 22-h duration. The artificial motion generated 5 min of motion every 30 min. From the 264 5-min time segments, 44 motion and 220 non-motion cases were present. Accordingly, 43 videos recorded actual motion when motion was generated. One motion was missed, and no video was created for it. The 220 5-min time segments did not create videos when there was no motion. This results in an accuracy of 0.99, compared to previous attempts which achieved 0.95 [30]. In the outdoor trial, the device self-restarted twice due to a flat battery. Over the 19 days, an unknown error occurred only once, where the camera froze and restarted. Random data were selected over the 19 days where captured videos showed no animal movement; however, during low light operation, footage of animals occurred.

As power consumption of the camera trap occasionally ran the battery below the RPi’s voltage input threshold, shutdown occurred 2.5% of the time while operating off of a 100-W solar panel. Future power optimization should mitigate this issue. The majority of the CPU’s cores ran at full capacity, meaning thermal dissipation was an issue, where the heatsink was unable to sink enough heat into the aluminium enclosure. It was noticed that many videos captured during the day were of shadows moving rather than animals. These false positives are inevitable when aiming to record small animals, and currently require post-processing to eliminate, although a future design will include such processing on the camera. It should be mentioned at this point that even with these false positives, the data load is still significantly reduced compared to a continuously recording system. A future revision is currently being designed to accommodate the majority of the issues listed with the current camera trap. We are now developing a robust shadow detection algorithm that performs better with variations within images such as shadows.

**Figure 9:**
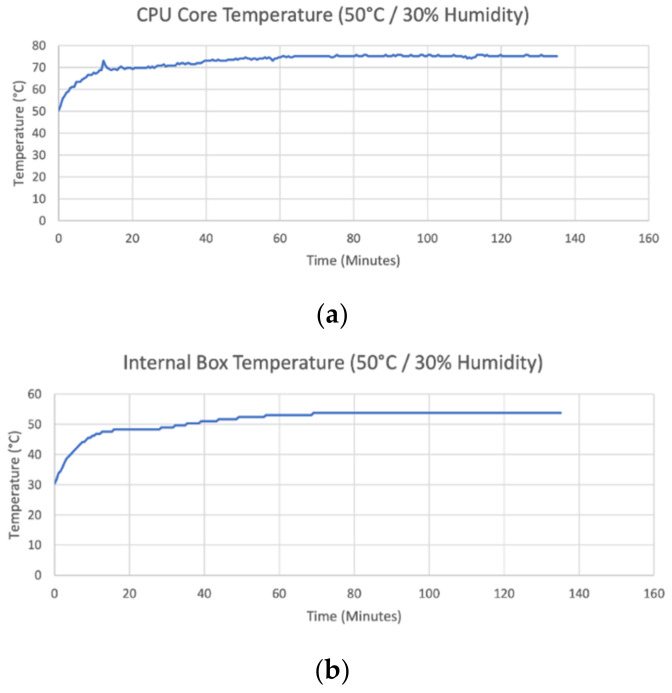
Experiment 3 results from environmental chamber testing of the camera trap. (**a**) CPU temperature with environmental chamber at 50 °C and 30% humidity. (**b**) Internal box temperature with environmental chamber at 50 °C and 30% humidity.

Experiment 3 tests are shown in Figure 9. These results show the camera trap’s ability to thermally dissipate heat from the CPU under experimental conditions using an environmental chamber. During the experiment, the CPU reduced its speed from 1.4GHz to 1.2 GHz to prevent overheating.

### 4.1. In-Field Results

In-field results created more than 500 GB of data total from all the camera traps, consisting of 85,870 video segments, each of a few seconds duration. Only 27 of the total 36 cameras recorded data during deployment. 12 cameras captured data for more than 25 days; the rest failed after 1 to 2 weeks. The majority of cameras stopped recording after 66 detections per day and rebooted at midnight every night. The camera trap includes a software feature to remove any potential hidden software bugs by clearing all the internal memory and variables to support continuous operation. Our tests were done during the Australian summer, when temperatures varied between over 20 °C during the nights and over 40 °C during the days. Failure of cameras is suspected to be from overheating, as some days recorded temperatures over 40 °C during an Australian summer. This reinforces the importance of using a robust enclosure or a cooling option in harsh environments.

After manually labelling 6135 video segments (7.1% of the total video segments), we encountered 1548 video segments containing an animal, including very small insects and non-identifiable animals. Therefore, ~25% of footage contained an animal, mostly small insects. Furthermore, 29 video segments contained a mammal, reptile, amphibian, or bird, accounting for 0.49% of the 6135 labelled segments. Including video segments of large arthropods brought the total to 423 labelled video segments, accounting for 6.9% of labelled video segments.

A total of 423 video segments successfully captured ectotherms, with 17 video segments of reptiles (0.3%), 5 video segments of amphibians (0.1%), and 401 video segments of large arthropods (6.5%). The majority of captured footage of ectotherms occurred during the night.

These results demonstrate the system’s ability to successfully record ectotherms and endotherms; however, this came with the trade-off that many false positives were also recorded (75%) when wind caused moving shadows, leaves, or grass within the frame. Methods to further automate data reduction are in development using machine (deep) learning, which will enable this volume of data to be cost-effectively processed.

### 4.2. Cost

Table 4 demonstrates the cost of the camera trap, with a total cost of A$1343.03 (including labour) for a single device.

## 5. Limitations and Future Work

**Figure 10:**
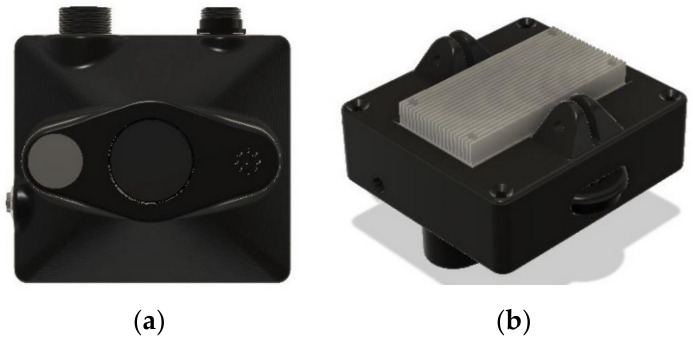
Future Camera Trap. (**a**) Front view. (**b**) Top view.

The next revision of the camera trap is currently being developed with an entire redesign of the enclosure. This redesign provides for better thermal performance and operation of the camera trap in a much smaller case for ease of transportation. The next camera trap revision prototype is depicted in Figure 10. A key limitation is maintaining power to the cameras. While mild operational performance was achieved in low open woodlands in mid-summer, short days and higher tree canopy cover will present challenges for the current power set up, requiring increased battery storage and solar panel area. Power consumption is to be further optimized with a future camera trap, which is currently in the works. A second key limitation is on-board processing power. The RPi was pushed to capacity, and it then required a low-demand video format to operate consistently. This design choice was essential to limit costs to a price range that makes field deployment affordable. New CPUs are now available that could be included to improve on-board processing power, such as the Arduino MKR Vidor 4000. These would enable video with no animals present to be deleted, reducing data transmission and storage needs. Ultimately, on-board species-recognition may enable tiny amounts of data to be routinely transmitted by satellite links, making autonomous monitoring possible throughout remote parts of the world.

## 6. Conclusions

This paper demonstrated a novel and effective new camera trap with in-field testing showing viability for the recording of ectotherms and endotherms in motion. Motion detection algorithms were successfully used to record animals present within video frames. Lab testing with artificial motion achieved an accuracy of 0.99. From the field testing, it was observed that the ingress protection of the enclosures was operating correctly, with no water, dust, or ant ingression into the enclosures. Using an AI algorithm to evaluate and then store or further process videos has enormous potential to completely overcome limitations of standard camera traps that use passive infrared triggering methods. AI methods can expand automated wildlife surveys to reptiles, amphibians, and invertebrates, and do away with issues of trigger speed, which can limit detection of fast-moving animals when slow [32]. With AI triggering only beginning to surface, there are many exciting future possibilities for powerful new monitoring tools capable of contributing to improved monitoring and conservation of the world’s biodiversity.

## Figures and Tables

**Table 1:** Average and peak power and current consumption rates of the camera trap system during good light conditions.

Consumption	*Average*	*Peak*
Power (W)	2.65	3.758
Current (A)	0.221	0.313

**Table 2:** Average and peak power and current consumption rates of the camera trap system during low light conditions.

Consumption	*Average*	*Peak*
Power (W)	4.13	5.208
Current (A)	0.344	0.434

**Table 3:** Confusion Matrix results on True Positives, True Negatives, False Positives, False Negatives, and Accuracy.

Confusion Matrix	Predicted: No Motion	Predicted: Motion	Total:
Actual: No Motion	True Negative: 220	False Negative: 0	220
Actual: Motion	False Positive: 1	True Positive: 43	44
Total:	221	43	264
Accuracy			0.99

**Table 4:** Main items and their associated costs.

Item	*Parts*	*Price*
Electronic Components	94	A$316.50
PCB Manufacturing	4	A$10.00
Solar	9	A$336.00
Fasteners	20	A$24.97
3D Printing	3	A$194.07
Labour	1	A$460.80
Total	131	A$1343.03

## Data Availability

Not applicable.
